# Muscle Derived Stem Cells Stimulate Muscle Myofiber Repair and Counteract Fat Infiltration in a Diabetic Mouse Model of Critical Limb Ischemia

**DOI:** 10.4172/2157-7633.1000370

**Published:** 2016-12-26

**Authors:** J Tsao, I Kovanecz, N Awadalla, R Gelfand, I Sinha-Hikim, RA White, NF Gonzalez-Cadavid

**Affiliations:** 1 Department of Medicine, Charles R. Drew University of Medicine and Science, Los Angeles, CA, USA; 2 Department of Surgery, Harbor-UCLA Medical Center and Los Angeles Biomedical Research Institute, Torrance, CA, USA; 3 Department of Urology, David Geffen School of Medicine at UCLA, Los Angeles, CA, USA

**Keywords:** MDSC, Follistatin, Molsidomine, Nitric oxide, Type 2 diabetes

## Abstract

**Background:**

Critical Limb Ischemia (CLI) affects patients with Type 2 Diabetes (T2D) and obesity, with high risk of amputation and post-surgical mortality, and no effective medical treatment. Stem cell therapy, mainly with bone marrow mesenchymal, adipose derived, endothelial, hematopoietic, and umbilical cord stem cells, is promising in CLI mouse and rat models and is in clinical trials. Their general focus is on angiogenic repair, with no reports on the alleviation of necrosis, lipofibrosis, and myofiber regeneration in the ischemic muscle, or the use of Muscle Derived Stem Cells (MDSC) alone or in combination with pharmacological adjuvants, in the context of CLI in T2D.

**Methods:**

Using a T2D mouse model of CLI induced by severe unilateral femoral artery ligation, we tested: a) the repair efficacy of MDSC implanted into the ischemic muscle and the effects of concurrent intraperitoneal administration of a nitric oxide generator, molsidomine; and b) whether MDSC may partially counteract their own repair effects by stimulating the expression of myostatin, the main lipofibrotic agent in the muscle and inhibitor of muscle mass.

**Results:**

MDSC: a) reduced mortality, and b) in the ischemic muscle, increased stem cell number and myofiber central nuclei, reduced fat infiltration, myofibroblast number, and myofiber apoptosis, and increased smooth muscle and endothelial cells, as well as neurotrophic factors. The content of myosin heavy chain 2 (MHC-2) myofibers was not restored and collagen was increased, in association with myostatin overexpression. Supplementation of MDSC with molsidomine failed to stimulate the beneficial effects of MDSC, except for some reduction in myostatin overexpression. Molsidomine given alone was rather ineffective, except for inhibiting apoptosis and myostatin overexpression.

**Conclusions:**

MDSC improved CLI muscle repair, but molsidomine did not stimulate this process. The combination of MDSC with anti-myostatin approaches should be explored to restore myofiber MHC composition.

## Introduction

Critical Limb Ischemia (CLI) is a devastating disease that mainly affects patients with Type 2 Diabetes (T2D) who are also obese. Such patients have a high risk of amputation and post-surgical mortality, and there is no effective medical treatment [1–3]. T2D, obesity, and metabolic syndrome are at epidemic proportions in the USA [4,5], and affect minorities disproportionately. Moreover, complications in minorities are more severe. One of the most serious complications is peripheral artery disease, and specifically it’s most severe form, CLI [1–3]. In this condition, blood flow is insufficient to maintain tissue viability, causing extreme chronic pain, non-healing ulcers, or gangrene in the leg/foot. There is often neuropathy and/or necrosis of the skeletal muscles, arteries and other tissues. CLI occurs in the US at approximately 300,000 cases/year. It is the leading cause of lower limb amputation. Due to CLI, the risk of amputation is 40 times greater in the diabetic population [1].

Ischemic injury in normal tissue is characterized by a revascularization compensatory response including angiogenesis and arteriogenesis, but this response is defective in CLI [6]. This may result in extensive muscle necrosis [7]. Surgical, endovascular revascularization, and medical approaches intended to stimulate angiogenesis are of limited therapeutic efficacy [2,8]. Therefore, CLI is an important subject for the search for novel vascular regeneration and muscle repair therapies. Stem cell implantation has been tried in animal models of ischemia using various kinds of cells, the most promising in terms of revascularization being Bone Marrow Mesenchymal Stem Cells (BMMSC), Adipose Tissue Derived Stem Cells (ATDSC), and Endometrial Regenerative Cells (ERC) [9–11]. However, few studies have been carried out in true T2D-CLI models, and none involving pharmacological modulation of stem cell stemness and differentiation.

Therapies involving stem cell implantation have been tried in human subjects. Although no serious side effects have been observed, angiogenesis was limited and the repair of muscle damage was not defined. Adding to the therapeutic challenges, autologous stem cell isolation is too invasive for CLI patients. Muscle Derived Stem Cells (MDSC), isolated from the skeletal muscle [12] have not been tested, but trials with other stem cells such as BMSC and ATDSC in diabetic patients are on-going or completed [13], using defined experimental outcomes such as, pain relief, walking distance, and wound healing, as opposed to the need for amputation. No serious side effects have been observed. Although some of these outcomes included modest improvements, and there may have been some advances in the underlying defect in angiogenesis, the possible reduction in necrosis or apoptosis of the skeletal muscle and repair of damaged myofibers were not reported.

A major hurdle for Stem Cell (SC) therapy in CLI associated with T2D, is the damaging diabetic tissue environment that may impair the functioning of implanted stem cells, just as it damages pre-existing tissue. In addition, this milieu may inhibit the survival and differentiation of implanted stem cells, as well as the underlying tissue, making it refractory to stem cell repair. The same toxic conditions may also limit the effectiveness of endogenous stem cells and their recruitment. One of the strategies to overcome these hurdles is the pharmacological modulation of stem cells in a way that restores their “stemness” in the context of the diabetic milieu in concert with methods to repair the damaging environment in the tissues.

Stimulators of the NO/cGMP pathway may fulfill both criteria. First, these NO and cGMP stimulating factors modulate stem cell differentiation [14–17]. Second, they stimulate blood flow and angiogenesis [18,19], as well as muscle repair, mainly through satellite cell fusion [20–23]. Specifically, NO, mainly from endothelial Nitric Oxide Synthase (eNOS) and acting through cGMP production, is essential for revascularization of the ischemic hindlimb, by acting as a vasodilator and by inducing VEGF, fibroblast growth factor, and urokinase-type plasminogen activator [24,25]. The NOS substrate L-arginine, eNOS overexpression, and phosphodiesterase 5 inhibitors (PDE5i) stimulate angiogenesis in animal models. Similar effects are exerted by NO donors and PDE5i on ischemia-reperfusion injury, and in muscle injury unrelated to T2D [26–32]. However, only one report deals with molsidomine in acute limb ischemia/reperfusion, but not CLI [32], and none of these studies was carried out in conjunction with stem cells.

The current study employed a T2D mouse model of severe CLI to address the combined effects of muscle ischemia and the diabetic milieu. We tested the efficacy of MDSC in inducing repair in T2D ischemic muscle tissue. We also tested the potential stimulation of this process by pharmacological modulation using the long acting nitric oxide generator, molsidomine. Finally, we evaluated the effects of MDSC implantation on the expression of myostatin, the main inhibitor of muscle mass growth and inducer of lipofibrosis [12,33,34], which is also involved in cardiac ischemia and its effects on the skeletal muscle [35].

## Methods

### MDSC isolation

Primary cultures of Muscle Derived Stem Cells (MDSC) were established with the preplating procedure described by Huard’s group [36], and extensively used by our group [12,37–39]. Briefly: Wild-type 12 to 16 week old control mice (C57BL/6J), referred to here as WT (Jackson Laboratories, Bar Harbor, ME, USA), were used as the source of MDSC. Hind limb muscles were dissociated using collagenase XI, dispase II, and trypsin. After filtration through 60-μm nylon mesh and pelleting, the cells were suspended in Plating Medium (PM), containing Dulbecco Modified Eagle Medium (DMEM), with 10% Fetal Bovine Serum (FBS), 20% horse serum, and 0.5% chick embryo extract (US Biological, Marblehead, MA, USA). Cells were added to a collagen I coated flask for 1 hour and non-adherent cells were collected and transferred to a second collagen I coated flask for an additional 2 hours. This process was followed by sequential daily transfers of non-adherent cells for 4 more days. Adherent cells at each stage were named preplate 1–6 (pP1–pP6). The pP6 is the cell population containing MDSCs, which were expanded by routine passage for experimental use (passages 14 to 28) in uncoated culture flasks with growth medium (DMEM with 20% FBS). Sca1+ cells were selected with Sca-1 (Ly6A) antibody-coated immunobeads (Milteni, Auburn, CA, USA). The full stem cell marker description of MDSC is available [40].

### Animal treatments

The T2D (db/db) mice were treated according to National Institutes of Health (NIH) regulations with an Institutional Animal Care and Use Committee-approved protocol. At 16 weeks of age, CLI was induced by severe Unilateral Femoral Artery Ligation (UFAL), leaving little or no collateral irrigation. The femoral artery was exposed and dissected from the vein and nerve, and two ligations were placed between the junction of the circumflex and the common iliac artery, then the ligated section was excised [41,42].

MDSCs were labeled with the nuclear fluorescent stain 4′, 6-Diamidino-2-Phenylindole (DAPI). Immediately following the UFAL procedure in the T2D mice, MDSCs were implanted aseptically in the surgically exposed gastrocnemius, and one day later molsidomine or saline was given as below. Group treatments were as follows (n=8/group): 1) Untreated, diabetic/UFAL, injected with 10 μl of vehicle (saline) in the UFAL leg (UT); 2) MDSC-alone, diabetic/UFAL, injected as in #1 but with 106 MDSC in total divided into 4 injections in 10 μl total volume (SC) [36]; 3) Molsidomine alone, diabetic/UFAL, daily IP injection of Molsidomine, 5 mg/kg/day (Mol); 4) MDSC/Molsidomine, diabetic/UFAL: as #2, but added daily IP injection of Molsidomine, 5 mg/kg/day (SC+Mol). Molsidomine treatment was carried out for 21 days. Non diabetic mice with the same genetic background (C57BLKS/J) and not subjected to UFAL or treatments were used as reference (ND-UT).

At the end point of the experiment, leg preservation and limb loss/motion/ischemia were determined. Following sacrifice, gastrocnemius muscles were excised from the affected limb. Tissues were divided for: a) paraffin embedding and cutting of 5 μm sections, b) freezing and storage at −80°C for western blots, and C) freezing medium embedding and cryosectioning at 10 um for Oil Red O staining. Blood was collected from the heart.

### Central nuclei, fibrosis, and fast myofibers

Samples of the gastrocnemius were embedded in paraffin and used for obtaining tissue sections that were subjected to histochemical Harris hematoxylin/eosin staining for detecting nuclei [12] and Masson trichrome staining for collagenous connective tissue fibers [12]. Myosin heavy chain type II (MHC-2) myofibers were detected by immunohistochemistry with primary antibody for MHC (fast) (anti-mouse MHC monoclonal, 1:400; Abcam). The sections were viewed under an Olympus IX71 inverted microscope coupled to a Leica digital bright-field light microscope/VCC camera. The histochemical staining was quantitated by image analysis using ImagePro-Plus 5.1 software (Media Cybernetics, Silver Spring, MD, USA). Images were corrected for background and integrated optical density (IOD, area × average intensity) was calculated. Central nuclei were counted.

### Apoptosis by TUNEL

In situ detection of cells with DNA strand breaks was performed on paraformaldehyde-fixed, paraffin-embedded muscle sections by the terminal deoxynucleotidyltransferase-mediated deoxyuridine triphosphate nick end labeling (TUNEL) technique, using an ApopTag-peroxidase kit (Chemicon International, Inc., San Francisco, CA) [37,43]. Counting of terminal deoxynucleotidyltransferase-mediated deoxyuridine triphosphate (DNT-DUT) nick end labeling-positive nuclei was carried out using an American Optical Microscope with a 40X objective and a pair of 10X eyepieces. A square grid fitted within one eyepiece provided a reference of 62,500 μm^2^. The rate of muscle cell apoptosis was expressed as the percentage of the terminal (DNT-DUT) nick end labeling-positive apoptotic nuclei per total nuclei (apoptotic plus nonapoptotic) present within the reference area.

### Determination of fat accumulation

Samples of the gastrocnemius were subjected to tissue freezing medium embedding, freezing and cryosectioning which were used for Oil Red O staining for detection of fat droplets [12]. Tissue sections were washed in 1 × PBS, fixed in 2% paraformaldehyde and stained with 0.5% Oil Red O (Sigma Chemical Co., Saint Louis, MO) for 15 min. For quantitative analysis of Oil Red O retention in tissue sections, extraction was accomplished using 1 ml of 4% Igepal CA-630 (Sigma) in isopropanol, and absorbance was measured by spectrophotometry at 520 nm.

### Protein analysis by western blot

Muscle tissues were homogenized in boiling lysis buffer; 1% Sodium Dodecyl Sulfate (SDS), 10 mM Tris buffer at pH 7.4 and 1X protease inhibitors. The homogenate was clarified by centrifugation at 16,000 g for 5 minutes, following which 20 μg protein samples were electophoresed through Bio-Rad Mini-Protean TGX 4–15% gels and transferred to PVDF membranes. Blots were probed with primary antibodies: (a) Oct 4 (anti-rabbit Oct 4, polyclonal, 1:500; BioVision, Inc., Milpitas, California, U.S.A.) [12]; (b) ASMA (anti-human ASMA, monoclonal, 1:1,000; Calbiochem, Billerica, Massachusetts, USA) [12]; (c) Calponin I (anti-mouse calponin 1, monoclonal, 1:500; Santa Cruz Biotechnology, Dallas, Texas, U.S.A) [12]; (d) VEGF (anti-mouse VEGF, monoclonal, 1:500; Santa Cruz Biotechnology) [43,44]; (e) Von Willebrand (anti-mouse Von Willebrand, polyclonal, 1:500; Abcam, Cambridge, Massachusetts, U.S.A.) [45]; (f) CD31 (anti-mouse CD31, monoclonal, 1:1000, Abcam) [46]; (g) BDNF (anti-mouse BDNF, rabbit monoclonal, 1:1000; Abcam) [38]; (h) MHC (fast) (anti-mouse MHC-II, monoclonal, 1:5000; Abcam) [12]; (i) myostatin (anti-mouse myostatin, rabbit polyclonal 1:1,000; Chemicon International Inc, Billerica, Massachusetts, U.S.A.) [12,47]; (j) follistatin (anti-mouse follistatin, rabbit polyclonal, 1:1000, Abcam) [48]; and (k) GAPDH (anti-mouse GAPDH, monoclonal, 1:5,000, Chemicon International).

Membranes were incubated with secondary anti-mouse IgG, horseradish peroxidase (HRP)-linked antibody (1:2,000; Cell Signaling Technology, Danvers, Massachusetts, U.S.A.) or anti-rabbit IgG linked to HRP (1:2,000, Amersham GE, Pittsburgh, PA, U.S.A.). Bands were visualized using luminol (SuperSignal West Pico; Chemiluminescent, Pierce, Rockford, IL, USA). For negative controls, the primary antibody was omitted.

### Statistical analysis

Values were expressed as mean ± standard error of the mean. Multiple comparisons were analyzed by single factor ANOVA, followed by post hoc comparisons with Tukey test. Comparisons between the treated groups and control UT group were made by unpaired two tail t-test using GraphPad Prism v5.01 statistical program (GraphPad Software Inc., San Diego, CA, USA). P<0.05 was considered statistically significant.

## Results

Intramuscular MDSC reduced mortality, stimulated stem cell number as well as myofiber central nuclei, and reduced both apoptosis and fat infiltration in the diabetic ischemic muscle, but molsidomine did not improve these effects

**Figure 1:**
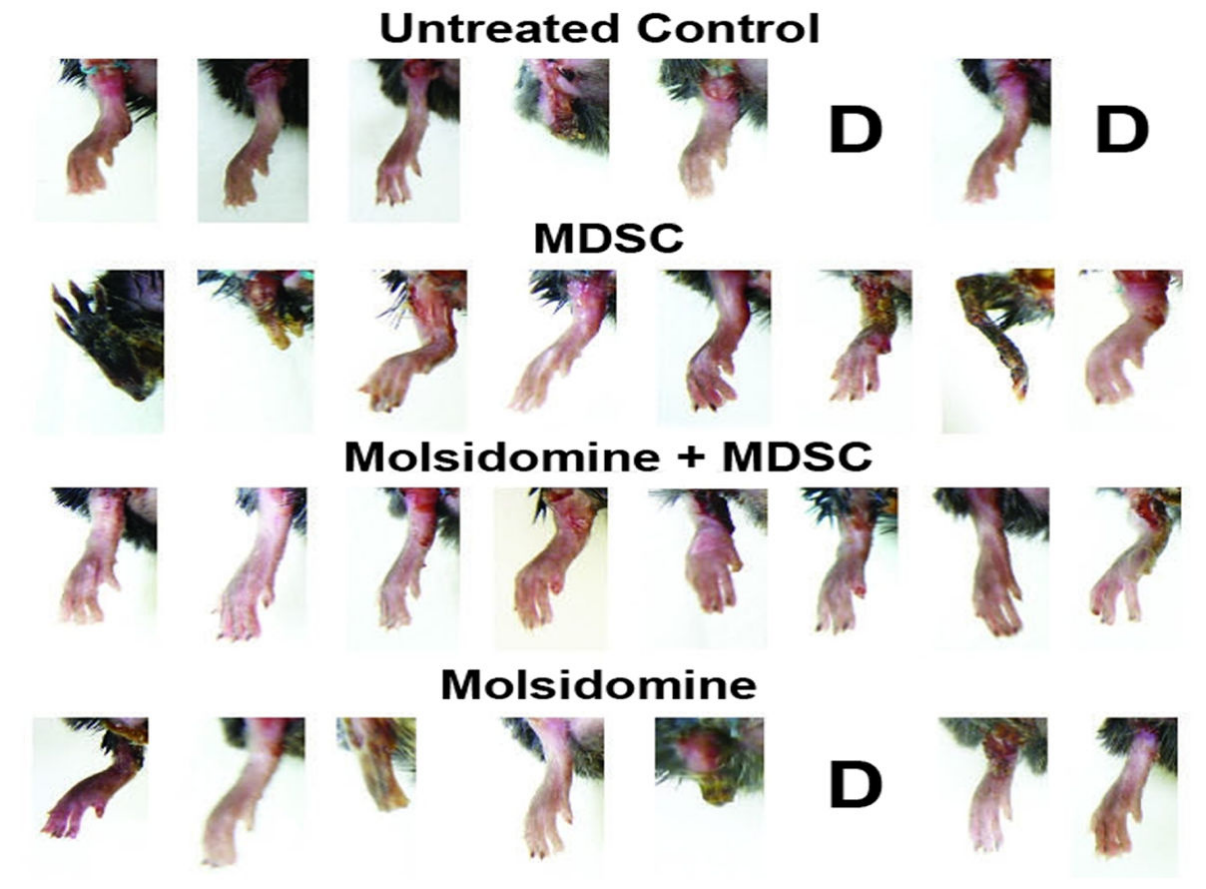
Implantation of MDSC in the ischemic limb muscle of the mouse model of diabetic CLI prevented mortality, but improvement in limb preservation was better when combined with intraperitoneal injection of molsidomine. MDSC (1 million cells) were implanted in several sites of the gastrocnemius in the CLI mice (n=8/group) immediately following a severe UFAL procedure. Animals were observed for 3 weeks before sacrifice. D: mouse died in period between 1 and 3 weeks. Limb loss is assumed to result at least in part from auto-amputation.

As expected, all the T2D mice showed considerable hyperglycemia of over 400 mg glucose/dl serum at sacrifice (4.5 months old), and were obese (35.4+/−4.1 g). ND-UT non-diabetic control mice had serum glucose of 100 mg/dl and were lean (27.3+/−1.4 g; n=8). No changes in serum glucose or body weight occurred with treatment. Within 3 weeks following UFAL 3 mice out of the original 32 had died and 3 suffered loss of one limb. Out of this total, the untreated UFAL group, referred to as UT (Figure 1), included one such limb loss and two deaths. The remaining 24 mice subjected to UFAL are designated in Figure 1 according to the therapeutic modalities that were attempted. MDSC implanted into the muscle immediately after UFAL prevented mortality. Supplementation of the MDSC treatment with molsidomine, but not molsidomine alone [38], improved limb preservation. However, the possibility of self-mutilation complicates the interpretation of leg loss.

**Figure 2:**
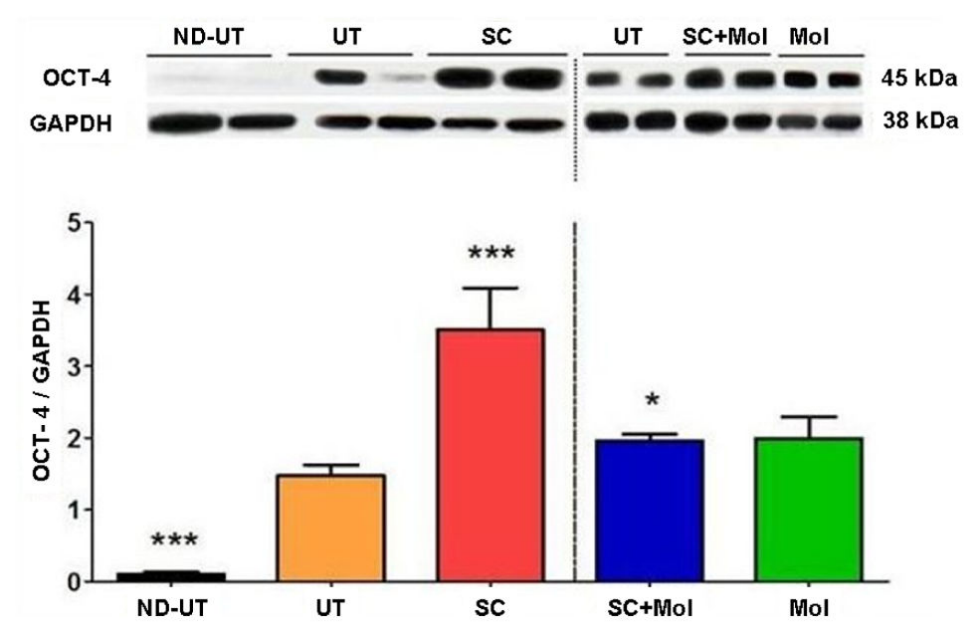
The treatment of the diabetic CLI muscle with MDSC stimulated the stem cell content, but supplementation with molsidomine counteracted this increase. Fresh ischemic or normal gastrocnemius tissue was subjected to quantitative western blot analysis of the stem cell marker OCT 4, correcting by GAPDH (bottom); two representative specimens per group are shown (top) (total n=8/group). ND-UT: non-diabetic, non-UFAL, untreated; UT: UFAL, untreated; SC: UFAL treated with MDSC alone; SC+Mol: UFAL treated with MDSC and molsidomine; Mol: UFAL treated with molsidomine alone. *p<0.05; ***p<0.001, Asterisks refer exclusively to comparisons performed against the untreated group (UT) subjected to UFAL as control. The absence of asterisks indicates non-significance against the UT group subjected to UFAL.

MDSC were visualized in frozen muscle tissue sections at completion by the presence of fluorescent DAPI+ nuclei. Quantitative western blot analysis was used to measure the expression of the stem cell marker Oct 4 in the UFAL-affected diabetic skeletal muscle, specifically its 45 kDa isoform corresponding to the stemness related Oct 4 protein localized in the nucleus [12]. Data were corrected by housekeeping gene expression (Figure 2). The data showed that in comparison to the non-diabetic control group not subjected to UFAL (ND-UT), the UT group showed a considerable increase of nuclear Oct 4, suggesting stem cell proliferation, or recruitment, triggered by ischemia as a compensatory mechanism. The implantation of MDSC (SC) increased the nuclear Oct 4 levels as expected, compared with the UT group. However, contrary to expectations the supplementation with molsidomine (SC+Mol) resulted in a considerable reduction in the MDSC effect to nearly the UT levels. Molsidomine alone (Mol) did not change Oct 4 level in comparison to the UT group.

**Figure 3:**
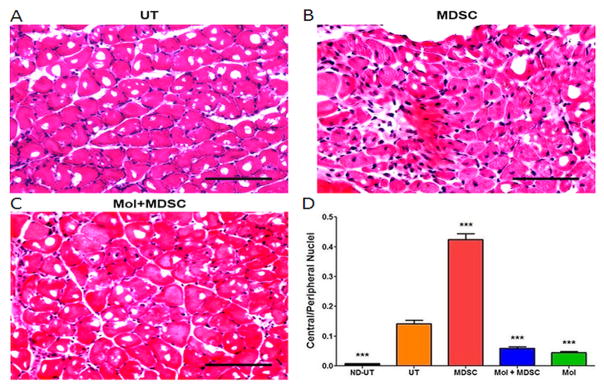
MDSC increased the early phase of myofiber repair in the CLI muscle but concurrent molsidomine reduced this effect. Fixed ischemic or normal gastrocnemius tissue was paraffin-embedded and tissue sections were used for hematoxylin/eosin staining and central and peripheral nuclei were counted by QIA (total n=8/group). A, B, and C: representative tissue section fields for the indicated groups. Magnification: 200X. Scale: 100 μm. D: quantitative data for central and peripheral nuclei (total n=8/group). ND-UT: non-diabetic, non-UFAL, untreated; UT: UFAL, untreated; MDSC: UFAL treated with MDSC alone; MDSC+Mol: UFAL treated with MDSC and molsidomine; Mol: UFAL treated with molsidomine alone; ***p<0.001. Asterisks refer exclusively to comparisons performed against the untreated group (UT) subjected to UFAL as control. The absence of asterisks indicates non-significance against the UT group subjected to UFAL.

Histochemical detection showed that the diabetic UT group had a much higher number of central nuclei in the myofibers than the ND-UT (non-UFAL) group, as can be expected from an endogenous repair response to the ischemic injuy (Figure 3). The MDSC induced a considerable increase in the number of central nuclei, indicating a stimulation of the myofiber repair process. In contrast, the Mol+MDSC and the Mol groups had an even lower number of central nucliei than in the UT, suggesting an unexpected inhibition of the myofiber repair process.

**Figure 4:**
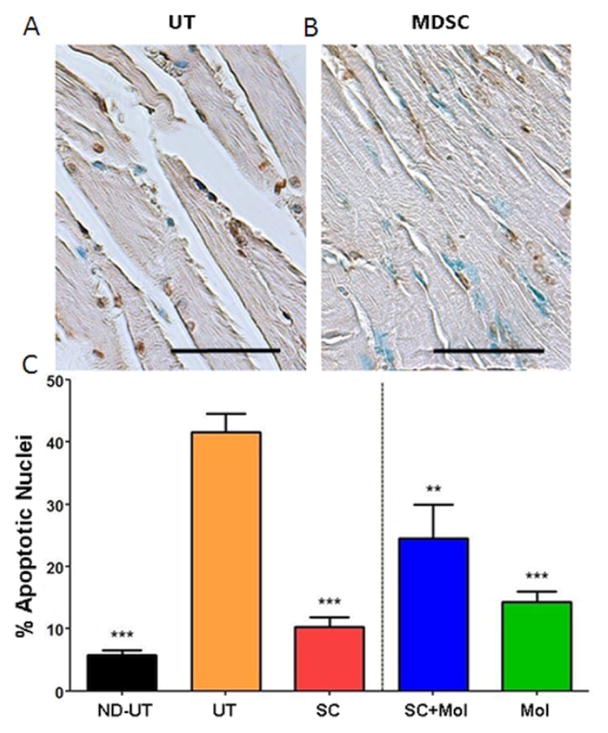
The treatment of the diabetic CLI muscle with MDSC reduced programmed cell death in the myofibers, but concurrent molsidomine was less effective. Similar tissue sections to those on Figure 3 were used for detection of apoptotic nuclei by the TUNEL reaction and counting by QIA. A and B: representative tissue section fields for the indicated groups. Magnification: 200X. Scale: 100 μm. C: quantitative data for the apoptotic index. (total n=8/group) ND-UT: non-diabetic, non-UFAL, untreated; UT: UFAL, untreated; SC: UFAL treated with MDSC alone; SC+Mol: UFAL treated with MDSC and molsidomine; Mol: UFAL treated with molsidomine alone. **p<0.01; ***p<0.001, Asterisks refer exclusively to comparisons performed against the untreated group (UT) subjected to UFAL as control. The absence of asterisks indicates non-significance against the UT group subjected to UFAL.

The diabetic ischemic muscle shows a considerable increase in apoptosis as estimated histochemically by the TUNEL technique, since the UT group has about 8-fold more apoptotic nuclei than the ND-UT (Figure 4). MDSC or molsidomine treatments by themselves were effective in reducing apoptosis to nearly the ND-UT levels. However, supplementation of MDSC with molsidomine reduced the antiapoptotic effects of MDSC, even if molsidomine by itself was anti-apoptotic.

**Figure 5:**
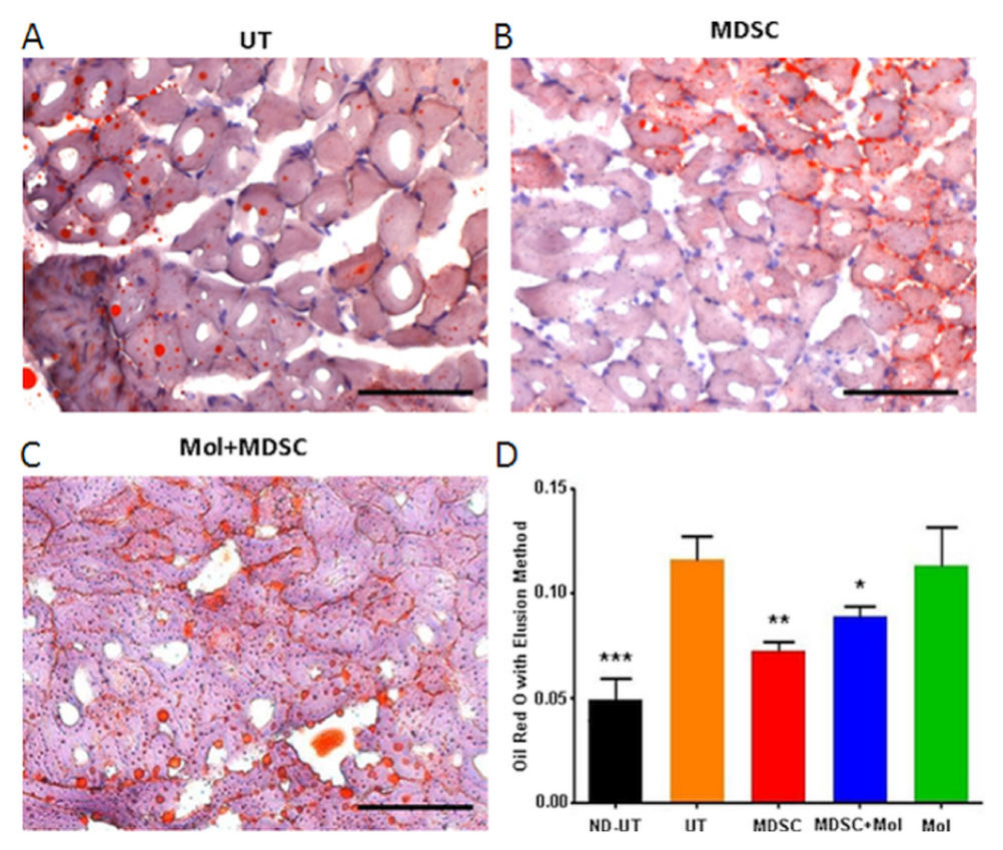
The treatment of the diabetic CLI muscle with MDSC reduced fat infiltration in and around the myofibers, but concurrent molsidomine did not improve efficacy. Frozen tissue sections from ischemic or normal gastrocnemius tissue were used for the detection of fat infiltration by Oil Red O staining, followed by elution and colorimetric estimation of the stain. A, B, and C: representative tissue section fields for the indicated groups. Magnification: 200X. Scale: 100 μm. D: quantitative data for the apoptotic index. (Total n=8/group) ND-UT: non-diabetic, non-UFAL, untreated; UT: UFAL, untreated; MDSC: UFAL treated with MDSC alone; MDSC+Mol: UFAL treated with MDSC and molsidomine; Mol: UFAL treated with molsidomine alone. *p<0.05; **p<0.01; ***p<0.001, Asterisks refer exclusively to comparisons performed against the untreated group (UT) subjected to UFAL as control. The absence of asterisks indicates non-significance against the UT group subjected to UFAL.

MDSC can also counteract fat infiltration in the diabetic ischemic muscle, as shown by the quantitative method of Oil Red O staining elution (Figure 5), applied to frozen tissue sections. The UT group had considerably higher intracellular fat than the ND-UT group, and fat was reduced in the SC group, but less in the MDSC+Mol group, and not affected in the Mol group, indicating that MDSC implantation acted against adipose degeneration in the diabetic ischemic muscle. However, in the CLI muscle, MDSC increased the relative area of inter-myofiber collagen compared to the total area, as measured by quantitative Masson trichrome staining on adjacent sections (not shown), as indicated by the SC group in comparison with the UT group (16.31+/−2.68 vs. 3.92+/−1.9) or to the ND-UT group (1.63+/−0.08). MDSC+Mol and Mol groups were not assayed. However, in the context of an ischemic muscle this apparently profibrotic effect may be functionally beneficial, through increased tensile strength.

### MDSC increase vascular and neural markers in the diabetic ischemic muscle, but molsidomine fails to stimulate these effects

**Figure 6:**
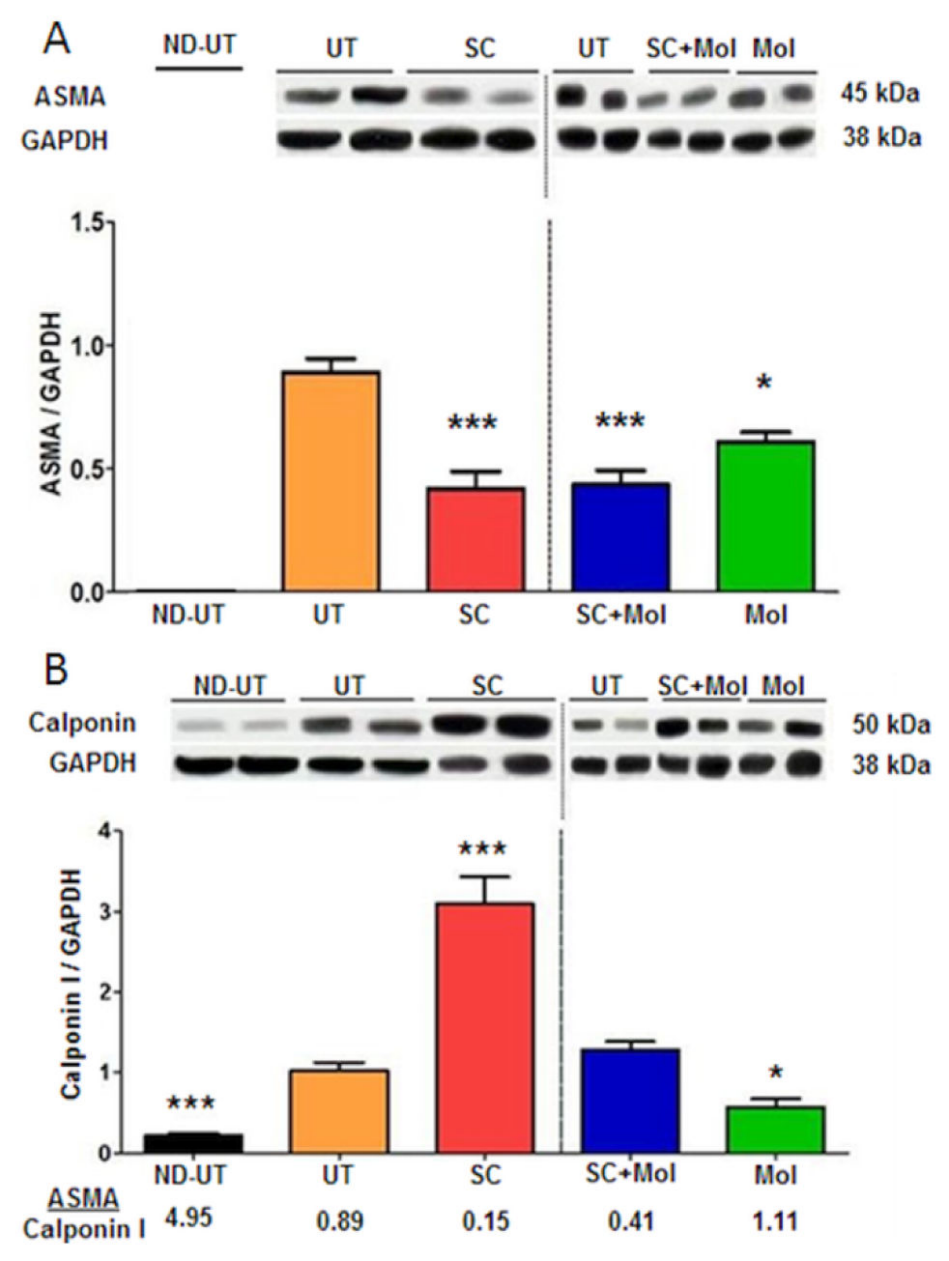
The treatment of the diabetic CLI muscle with MDSC increased the content of smooth muscle cells, presumably in the vasculature, and thus angiogenesis, but concurrent molsidomine reduced this effect. A: Fresh ischemic or normal gastrocnemius tissue was subjected to quantitative western blot analysis of the myofibroblast/SMC marker ASMA, correcting by GAPDH; two representative specimens per group are shown (total n=8/group). ND-UT is not shown B: as in the top panel, but for calponin 1, a mainly SMC marker. The higher the calponin 1/ASMA ratio higher the predominance of SMC over myofibroblasts. (total n=8/group). ND-UT: non-diabetic, non-UFAL, untreated; UT: UFAL, untreated; SC: UFAL treated with MDSC alone; SC+Mol: UFAL treated with MDSC and molsidomine; Mol: UFAL treated with molsidomine alone. *p<0.05; ***p<0.001, Asterisks refer exclusively to comparisons performed against the untreated group (UT) subjected to UFAL as control. The absence of asterisks indicates non-significance against the UT group subjected to UFAL.

To evaluate how these treatments affected angiogenesis, the content of smooth muscle and endothelium, presumably of vascular origin in the skeletal muscle, was evaluated. Vascular smooth muscle was determined by western blot by measuring the ratio between the expression of a marker protein for both smooth muscle cells and myofibroblasts, α-smooth muscle actin (ASMA), corrected by the predominant marker for smooth muscle cells, absent in myofibroblasts, calponin 1. As shown by ASMA staining, myofibroblasts, the cells involved in collagen deposition that accumulate during injury [47–49], were likely reduced in all treated groups, particularly in the SC group, in comparson to the UT group (Figure 6A), although in the presence of smooth muscle this conclusion must remain speculative. The ND-UT group was not assayed.

In contrast, the smooth muscle was specifically estimated, also by western blot, by calponin 1, and was found to be considerably increased in the SC group in comparison to the UT group (Figure 6B), but neither the SC+Mol nor the Mol groups had increased calponin 1 levels over the UT group. The most informative measure of the effects on smooth muscle vs myofibroblast content is the calponin 1/ASMA ratio. There was a 6-fold increase in the calponin 1/ASMA ratio in the SC group as compared to the UT group, but only a 2.2-fold increase in the SC+Mol group, suggesting that the SC+Mol combination was not efficacious in improving the stimulation of smooth muscle content by MDSC. This conclusion is supported by the fact that the Mol group showed no change in this ratio in comparison to the UT group.

**Figure 7:**
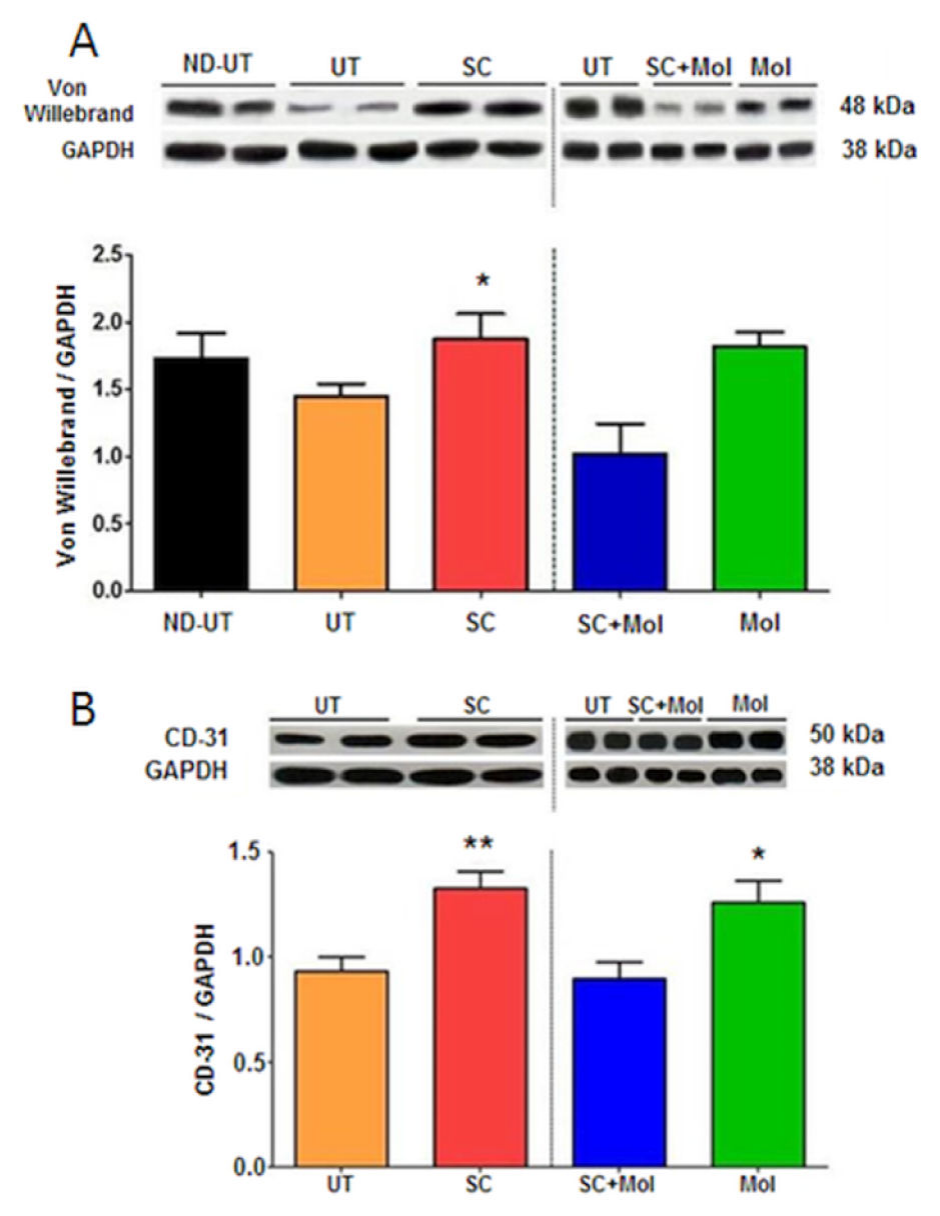
The treatment of the diabetic CLI muscle with MDSC increased the content of endothelial cells, presumably in the vasculature, and thus angiogenesis, but concurrent molsidomine abrogated this effect. A: fresh ischemic or normal muscle tissue was subjected to quantitative western blot analysis of the endothelial marker von Willebrandt factor, correcting by GAPDH; two representative specimens per group are shown (total n=8/group). B: as in the top panel, but for CD31; ND-UT was not assayed. (total n=8/group). ND-UT: non-diabetic, non-UFAL, untreated; UT: UFAL, untreated; SC: UFAL treated with MDSC alone; SC+Mol: UFAL treated with MDSC and molsidomine; Mol: UFAL treated with molsidomine alone. *p<0.05, Asterisks refer exclusively to comparisons performed against the untreated group (UT) subjected to UFAL as control. The absence of asterisks indicates non-significance against the UT group subjected to UFAL.

**Figure 8:**
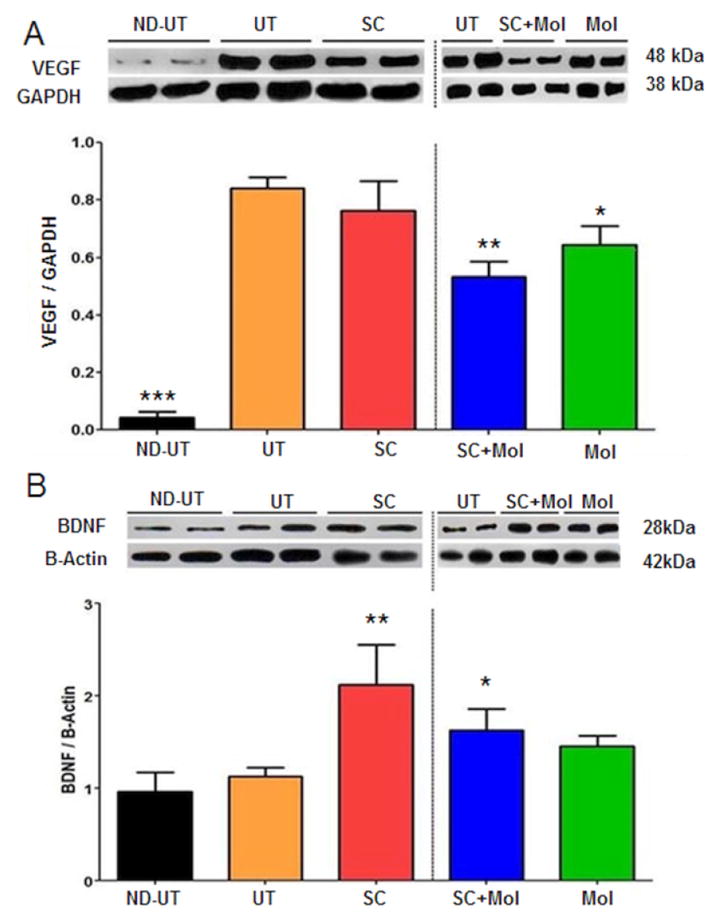
Treatment of the diabetic CLI with MDSC did not change the levels of the pro-angiogenic VEGF, but increased neurotrophic factor secretion, and concurrent molsidomine did not potentiate these effects. A: Fresh ischemic or normal gastrocnemius tissue was subjected to quantitative western blot analysis of VEGF, correcting by GAPDH; two representative specimens per group are shown (total n=8/group): B: a similar assay was performed for BDNF, correcting for beta actin ND-UT: non-diabetic, non-UFAL, untreated; UT: UFAL, untreated; SC: UFAL treated with MDSC alone; SC+Mol: UFAL treated with MDSC and molsidomine; Mol: UFAL treated with molsidomine alone. *p<0.05; **p<0.01; ***p<0.001, Asterisks refer exclusively to comparisons performed against the untreated group (UT) subjected to UFAL as control. The absence of asterisks indicates non-significance against the UT group subjected to UFAL.

In agreement with this stimulation of presumptive vascular smooth muscle, MDSC also stimulated endothelial cell content, presumably also in the muscle vascular system, as shown in western blots by higher Von Willebrand factor (Figure 7A) and CD31 (Figure 7B) expression levels. In these cases SC+Mol failed to improve the MDSC effect, although Mol by itself did result in upregulation. The expected very high expression of VEGF in the ischemic muscle was indeed observed in the UT group in comparison to the ND-UT group (Figure 8A), but, as for smooth muscle, SC did not increase VEGF further, and the other two treatments even reduced it. MDSC also stimulated the release of neurotrophic factors in the diabetic ischemic mouse, as shown by the western blot for Brain Derived Neurotrophic Factor (BDNF) expression (Figure 8B). Supplementation with molsidomine reduced this effect, instead of stimulating it as expected, and Mol alone did not change the expression.

The stimulation of myofiber repair by MDSC does not lead to the total recovery of the myofiber composition, and this may be due to the overexpression of myostatin induced by MDSC.

**Figure 9:**
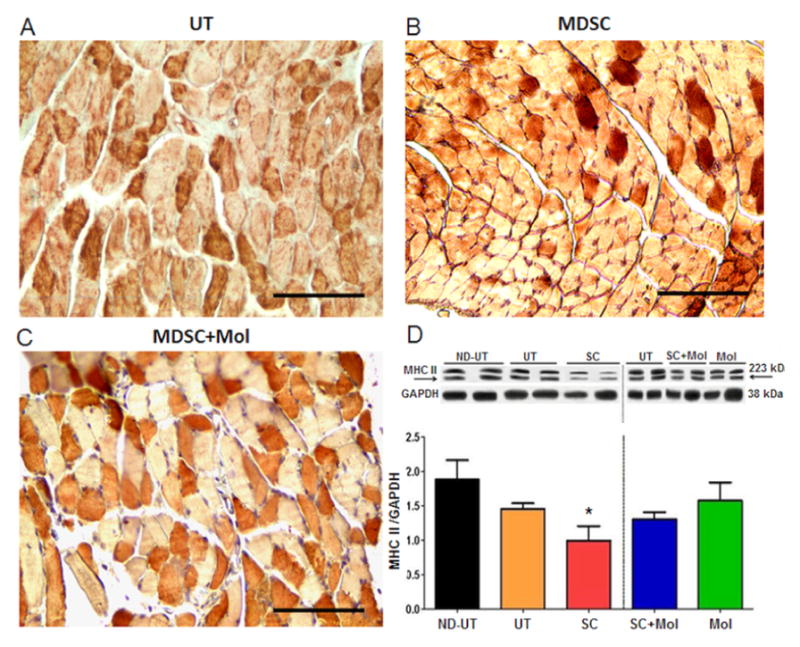
Despite the observed increase in early myofiber repair, the treatment of the diabetic CLI muscle with MDSC alone reduced the relative content of fast versus slow MHC myofibers content, and the same occurred with concurrent molsidomine. A, B, and C: Fixed ischemic or normal gastrocnemius tissue was paraffin-embedded and tissue sections were used for MHC-II staining and positive and negative myofibers were counted by QIA. Representative tissue section fields for the indicated groups are shown, but since the total n=4/group was insufficient values were not plotted. They confirmed the quantitative data determined by western blot. Magnification: 200X. Scale: 100 μm. D: fresh ischemic or normal gastrocnemius tissue was subjected to quantitative western blot analysis of MHC-II with a different antibody (see Methods), correcting by GAPDH; two representative specimens per group are shown (total n=8/group). ND-UT: non-diabetic, non-UFAL, untreated; UT: UFAL, untreated; SC: UFAL treated with MDSC alone; SC+Mol: UFAL treated with MDSC and molsidomine; Mol: UFAL treated with molsidomine alone. *p<0.05, Asterisks refer exclusively to comparisons performed against the untreated group (UT) subjected to UFAL as control. The absence of asterisks indicates non-significance against the UT group subjected to UFAL.

Figure 9 shows by immunohistochemistry and western blot that although there was the expected moderate decrease in the expression of MHCII, a marker of mature myofibers, in the diabetic ischemic muscle (UT vs. ND-UT), this was not compensated (and even diminished) in the SC group, despite the MDSC stimulatory effects on the number of central nuclei observed in Figure 3. SC-Mol, and Mol did not modify the expression.

**Figure 10:**
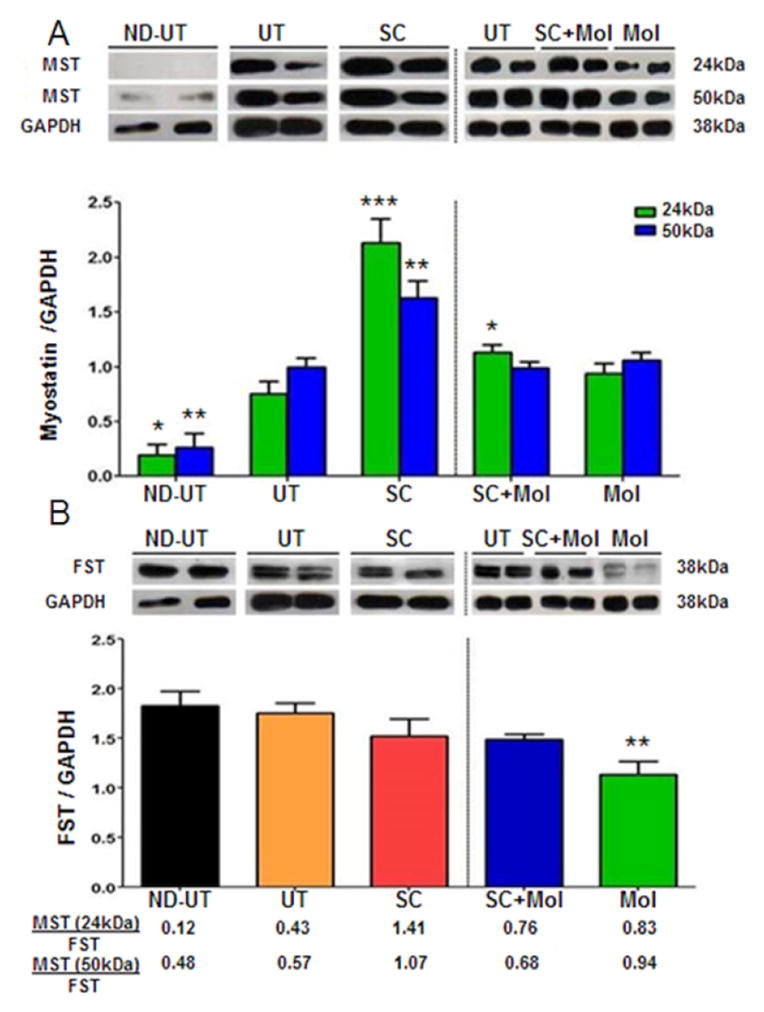
The treatment of the diabetic CLI muscle with MDSC or with concurrent molsidomine upregulated the muscle mass inhibitor and profibrotic factor myostatin, but did not change the levels of its antagonist, follistatin. A: fresh ischemic or normal gastrocnemius tissue was subjected to quantitative western blot analysis of myostatin, corrected by GAPDH; two representative specimens per group are shown (total n=8/group). B: as in the top panel, but for follistatin. The higher the myostatin/follistatin ratio the higher the profibrotic and muscle mass inhibition effects. ND-UT: non-diabetic, non-UFAL, untreated; UT: UFAL, untreated; SC: UFAL treated with MDSC alone; SC+Mol: UFAL treated with MDSC and molsidomine; Mol: UFAL treated with molsidomine alone. *p<0.05; **p<0.01; ***p<0.001, Asterisks refer exclusively to comparisons performed against the untreated group (UT) subjected to UFAL as control. The absence of asterisks indicates non-significance against the UT group subjected to UFAL.

In order to investigate a possible mechanism of interference with the repair capacity of the MDSC implanted in the diabetic ischemic muscle, we measured the levels of myostatin (also referred to as GDF-8) by western blot. Myostatin is well established to be a major inhibitor of striated muscle mass growth, and a pro-lipofibrotic factor [47,49]. The UT group shows overexpression of the myostatin 50 kDa and 25 kDa bands, representative of the glycosylated 375 amino acid monomer precursor protein and the dimer of the processed 110 amino acids protein, as compared to the ND-UT group (Figure 10A).

The SC group showed a considerable upregulation of myostatin levels induced by the MDSC implantation that would be expected to counteract the final MDSC repair of the myofibers, at least in terms of the observed MHC-II vs. MHC-I composition. The molsidomine supplementation in the SC+Mol group partially counteracted this myostatin over-expression, and this was more evident in the molsidomine alone group. The expression of follistatin, the main myostatin inhibitor is not affected in the UT or in the treated groups, except for a minor reduction in the Mol group (Figure 10B). Therefore, the ratio of myostatin to follistatin is higher than in the UT group not just in the SC group (over 2-fold) but also in the SC+Mol and Mol groups, indicating in the latter treatments some residual bioavailable myostatin not blocked by follistatin.

## Discussion

In this study, we investigated potential therapeutic effects of MDSC implantation in a mouse model of CLI in T2D. An experimental version of CLI was created using femoral artery ligation to induce severe ischemia in the hind limb. This study shows that implantation of MDSC into the gastrocnemius damaged by CLI prevented mortality, albeit with some necrosis, and in the ischemic muscle induced some of the expected beneficial effects, namely: a) increased stem cell number, the myofiber central nuclei ratio to the total nuclei within the myofibers that characterizes muscle repair, the content of smooth muscle and endothelial cells denoting a presumable angiogenic effect, and the expression of a key neurotrophic factor; and b) reduced the myofiber apoptotic index and fat infiltration. The stimulation of myofiber repair was partial, and did not lead to the complete restoration of the myofiber fast/slow composition. We assume that some of these pitfalls were probably due in part to the overexpression of myostatin induced by MDSC, which may result from stem cell implantation in the ischemic muscle, and may justify future studies with a concurrent anti-myostatin approach. We also observed that supplementation of MDSC with the NO donor molsidomine failed to improve on the beneficial myogenic or angiogenic effects of MDSC.

Very few studies in animal models of diabetic limb ischemia, and none in humans, have focused on the use of stem cells for repair of atrophic skeletal muscle. In particular, few studies deal with lipofibrotic degeneration as the main target of stem cell therapy. In earlier studies using mouse models, the implanted stem cells were mostly Endothelial Progenitor Cells (EPC) [50–52], Epidermal Progenitor Cells [53], or Human Neural Stem Cells [54]. Some studies dealt with the mobilization of endogenous EPC [55,56], and none used MDSC or ADDSC. There is also a paucity of studies on true T2D mouse models, specifically db/db [56,57], since most used streptozotocin-induced T1D models [50,51,53,55,56]. There are advantages in using a T2D db/db model, since the recovery from limb ischemia is less effective than in the T1D model [56], thus allowing better prediction of efficacy in human T2D, particularly in the severe ischemia that we induced by also blocking femoral artery collateral irrigation. Interestingly, auto-amputation (limb loss) was reported in the KK/AY T2D mouse [57] but no stem cells were used as therapy in this study.

None of the studies using stem cells in diabetic animal models of CLI focused on myofiber recovery in the affected limb, including one study in the rat [58]. Most were limited to stimulation of blood flow, angiogenesis, and/or revascularization by the injected stem cells, the conditioned medium, or the pharmacological agents triggering endogenous stem cell mobilization and recruitment [51–57,59]. There was upregulation or increased release of VEGF in some earlier studies [51,59]. In our study no such changes were observed upon MDSC implantation, in comparison with the untreated ischemic controls, even if smooth muscle and endothelial markers, presumably vascular, were indeed upregulated.

Our results with MDSC treatment do agree with the reduction in apoptosis in the ischemic muscle observed in two studies [50,53], and with the presence of fatty infiltration in ischemic muscle [56]. However, there are no related reports in the CLI/diabetes animal models that relate to our MDSC treatment data. In particular, we found that MDSC implantation stimulates increases in Oct 4 (stem cell content), central nuclei (early myofiber regeneration), and BDNF (neurogenesis). We also observed an increase in the calponin 1/ASMA ratio (angiogenesis, less fibrosis) and endothelial markers. Our study was conclusive in terms of MDSC alone reducing apoptosis, fat infiltration and myofibroblast content, but associated with higher collagen deposition.

The increase in myostatin and in the myostatin/follistatin ratio we observed in response to MDSC treatment is also novel, since no studies on either protein in the skeletal muscle after stem cell treatment of CLI in animal models or humans have been reported. The increase in myostatin does agree with similar changes we have found after treatment with MDSC of penile corporal fibrosis in T2D rats [39]. The fact that in our model, limb ischemia or T2D per se raised the myostatin compared with the non-diabetic/non FAL animals, is also of interest, since no related reports are available on this protein in the muscle in CLI models in the absence of stem cell implantation. However, it does agree with the increase of myostatin in the heart muscle induced by ischemia [35].

If our observations on the overexpression of myostatin are confirmed in other T2D/CLI animal models implanted with stem cells, it may suggest that such implantation may counteract at least partially the beneficial effects exerted by MDSC (or stem cells in general), and may lead to the alteration of the balance between slow and fast myofiber regeneration. In fact our observation that MDSC did not induce the recovery of the MHC-II positive myofibers in comparison to MHC-I myofibers may support this hypothesis, since we previously showed that myostatin expression is associated with lower content of MHC-II myofibers [60]. Moreover, myostatin upregulates slow but downregulates fast MHC isoforms, and is expressed at higher levels in the fast muscle myoblasts and myofibers than in the slow muscle counterparts, which implies an inhibitory role of myostatin for fast MHC expression [61]. It is also known that in patients with peripheral arterial obstructive disease the content of MHC II decreased with a higher grade of ischemia, consistent with an increased resistance to ischemia for MHC-I [62]. In addition, since myostatin is a lipofibrotic factor, it may partially counteract the beneficial reduction of fat infiltration in the muscle by MDSC, and be responsible for the moderate fibrosis observed using Masson trichrome.

Interestingly, follistatin-like-1 cDNA administration in a nondiabetic CLI mouse model promotes endothelial cell function and stimulates revascularization in response to ischemic insult through its ability to activate Akt-eNOS signaling [63]. Therefore, follistatin administration, or other antimyostatin approaches such as decorin, antibodies against myostatin, myostatin propeptide, or shRNA against myostatin [60,64–66] may be investigated as ancillary treatments to improve the effects of MDSC and other stem cell implantation for the treatment of CLI in T2D.

Molsidomine, either by itself or supplementing MDSC implantation, failed in our hands to exert the beneficial effects claimed in one other study on ischemia-reperfusion injury, incorrectly reported as CLI model [67], in this case in terms of reducing oxidative stress, necrosis and neutrophile infiltration, and thereby improving the histopathological score. The observed beneficial effects of molsidomine in that study may be due to the ischemia having been induced as an acute process caused by a very mild procedure (applying an elastic rubber band to the leg of a non-diabetic mouse), that could have minimized the damage, and to the extremely short period of observation (2 h) combined with the intravenous molsidomine injection. In our study, the ischemia is very severe and long-term, a true CLI model, and in T2D. It is therefore possible that the beneficial effects of molsidomine and other NO generators in skeletal muscle repair, such as reducing lipofibrosis in dystrophic muscle [16], sustaining long-term skeletal muscle regeneration by regulating the fate of satellite cells [20–23], or preventing ischemia-induced reperfusion injury in cutaneous and myocutaneous flaps, are not sufficient to counteract the profound injury caused by chronic severe CLI in the context of T2D.

## Conclusions

MDSC implantation into the ischemic limb muscle of diabetic mice reduced mortality, and in the muscle increased stem cell number, myofiber central nuclei, vascular and neural markers and reduced fat infiltration and apoptosis, possibly due to secretion of trophic factors. However, there was an incomplete generation of fast as compared to slow myofibers, which combined with the increase of interstitial collagen, was possibly due in part to the overexpression of myostatin induced by MDSC. Concurrent molsidomine was not effective. This suggests that the therapeutic use of MDSC for muscle repair in CLI in diabetes, or even stem cells in general, may be improved by concurrent anti-myostatin approaches. This may be studied in animal models of CLI to stimulate the stem cell repair capacity of the ischemic muscle.

## Abbreviations

ASMA: α-Smooth Muscle Actin
ATDSC: Adipose Tissue Derived Stem Cells
CLI: Critical Limb Ischemia
BDNF: Brain Derived Neurotrophic Factor
BMSC: Bone Marrow Stem Cells
MDSC: Muscle Derived Stem Cells
miRNA: MicroRNA
QIA: Quantitative Image Analysis
SMC: Smooth Muscle Cells
T2D: Type 2 Diabetes Mellitus
UFAL: Unilateral Femoral Artery Ligation
VEGF: Vascular Endothelial Growth Factor
